# Supplementary material for: Characterization of Non-Invasively Induced Post-Traumatic Osteoarthritis in Mice
Source: Antioxidants (Basel). 2022 Sep 9;11(9):1783. doi: 10.3390/antiox11091783 (PMC9495497; doi:10.3390/antiox11091783)
Supplement: Supplementary file 1 [file antioxidants-11-01783-s001.zip › antioxidants-1858401-supplementary.pdf]

Supplemental Figures and Legends

Supplemental Figure S1.

Macroscopic examination of knee joints. Blood clot formation on the knee joint in trauma group indicates severity and inflammation during joint damage. Note the disappearance of blood clot in PTOA group. (ML: mechanically loaded knee)

Supplemental Figure S2.

Detection of ROS-bioluminescence in knee joints of animals. L-012 is injected intraarticularly in animals after 1st, 4th and 6th mechanical loading session. Note that ROS detection is prominent only after 1st mechanical loading session. The ROS detection was not possible after successive mechanical loading sessions.

Supplemental Figure S3. Overview of DMA

A mouse tibia specimen mounted on the loading machine (Supplement Figure 3A), an example of dynamic mechanical analysis (DMA) to obtain phase angle and dynamic stiffness under oscillatory loading (-0.3 ± 0.1N) (Supplement Figure 3B).

Supplemental Figure S4. Modified OA Scoring and more histological sections

The knee joints were evaluated by H&E and safranin O staining in at least 2 serial mid-frontal sections. This histopathologic interpretation was conducted by at least three trained scientists (including a veterinary pathologist or an Orthopaedic surgeon) under blinded experiments, and the yielded data was utilized to determine the average value for OA and synovitis.

Tissue sections were graded using the osteoarthritis research society international (OARSI) and the Mankin scale with slight modifications. The advantage of these OA scoring system is that it allows scoring of the tissue sections based on the grade and stage of the disease's progression. Thus, a higher score represents a higher level of biological deterioration and disease extent. Therefore, it is a reliable method to differentiate between early- to late-stages of OA.

We further modified the procedure to assess the whole knee joint from an individual animal and sum up the average histological score from each representative group. Furthermore, the whole knee joint was graded by dividing it into four distinct regions as follows: medial femur (MF), medial tibia (MT), lateral femur (LF) and lateral tibia (LT). Each area was graded based on hypocellularity, Safranin-O staining, surface regularity and structure (Supplement Figure 4A). The mean from each area was calculated and summed up with other areas to yield the sum of the average histological score for each group of animals (Supplement Figure 4B and C). The detailed methodology to grade OA score has been outlined in the supplementary figures.

Synovitis in the medial and lateral knee joint was graded as described previously with slight modifications. Briefly, synovitis score was based on the enlargement of synovial lining, density of cells and inflammation. The highest score is 12 from combination of both part of knee joints (Synovitis in medial compartment (6) + lateral compartment (6)).

More hitological sections and scoring result in Figure 4D.

Supplemental Figure S1.

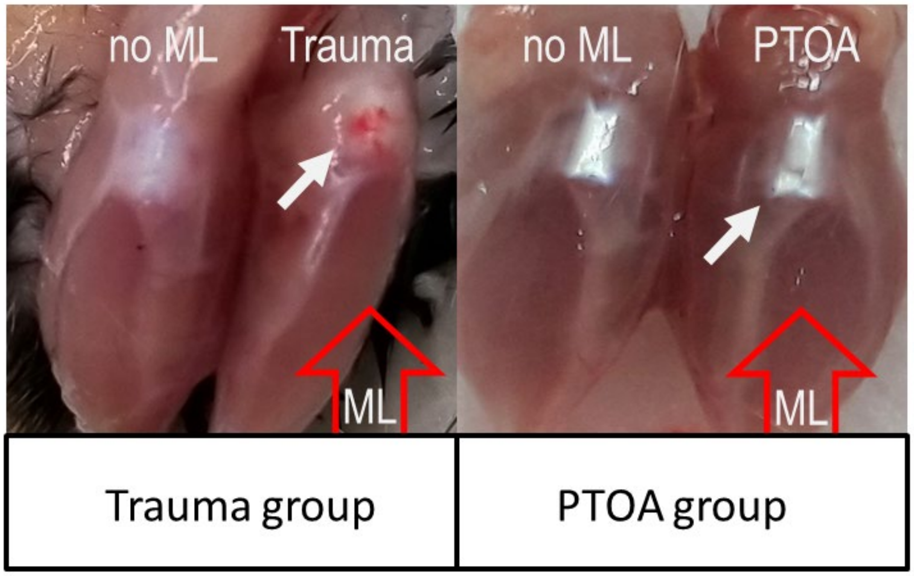

Supplemental Figure S2.

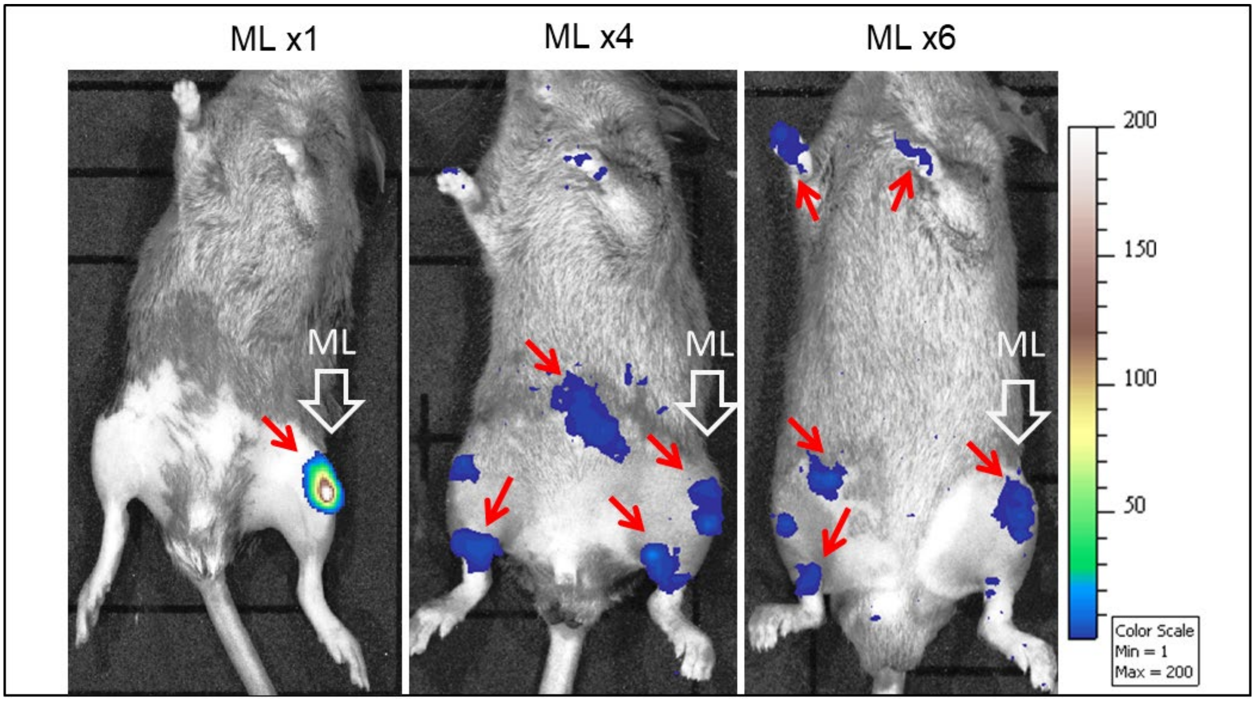

Supplemental Figure S3.

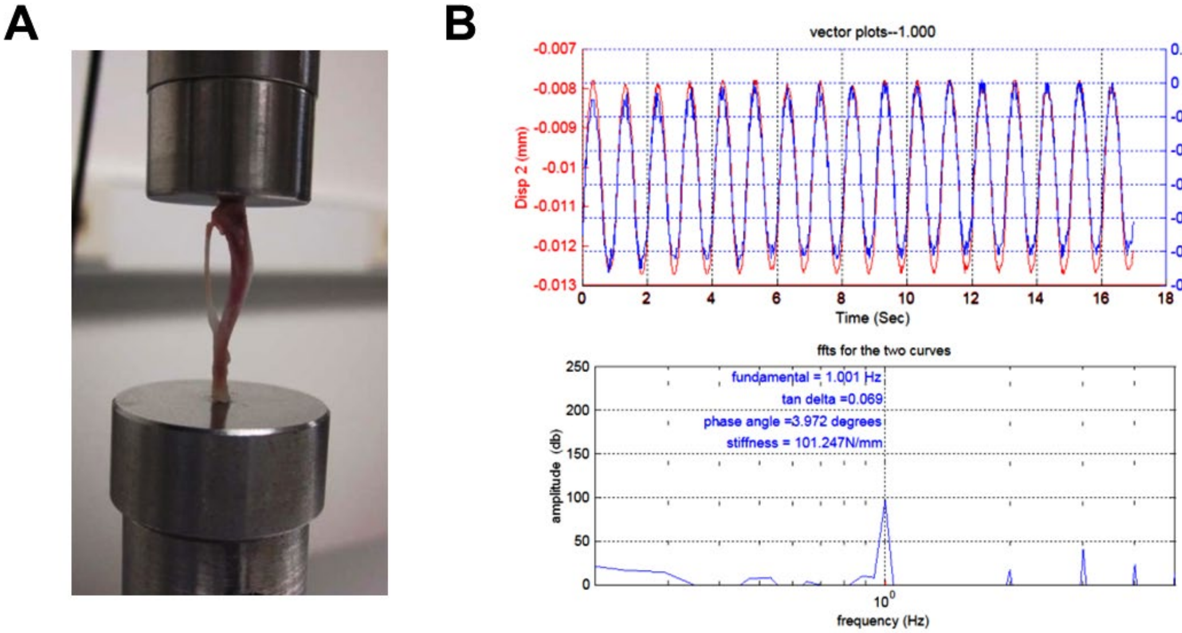

Supplemental Figure S4.

A.

CATEGORY		POINTS
Hypocellularity		
	Normal <5%	0
	Small decrease in chondrocytes <25%	1
	Large decrease in chondrocytes 25-75%	2
	No cells >75%	3
Safranin-O staining		
	Normal	0
	Slightly reduced	1
	Markedly reduced	2
	No metachromatin stain	3
Surface regularity		
	Smooth (>3/4)	0
	Moderate (1/2 to 3/4)	1
	Irregular (1/4 to 1/2)	2
	Severely irregular (<1/4)	3
Structure		
	Normal & Tidemark	0
	Deformation	1
	Fibrillation	2
	Fissures & Tide mark invaded	3
	Erosion	4
	Complete disorganization	5
Total maximum		14

B. Determine four areas for histopathological scoring

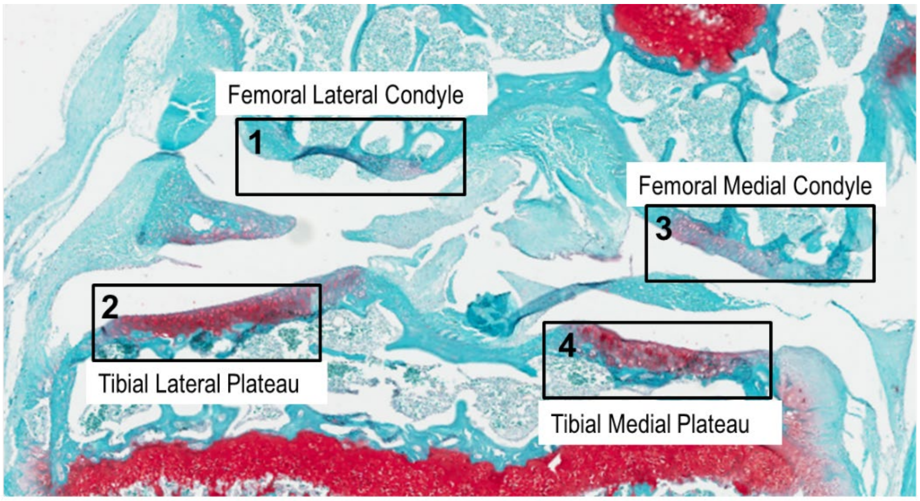

C. Calculation

<u>Histological Score Calculation:</u>

Sum of average histological score from each area per group

= average score of area 1 / group
+ average score of area 2 / group
+ average score of area 3 / group
+ average score of area 4 / group

Highest score = 56 (14x4)
Lowest score = 0 (0x4)

D. more histology sections and blinded scoring result

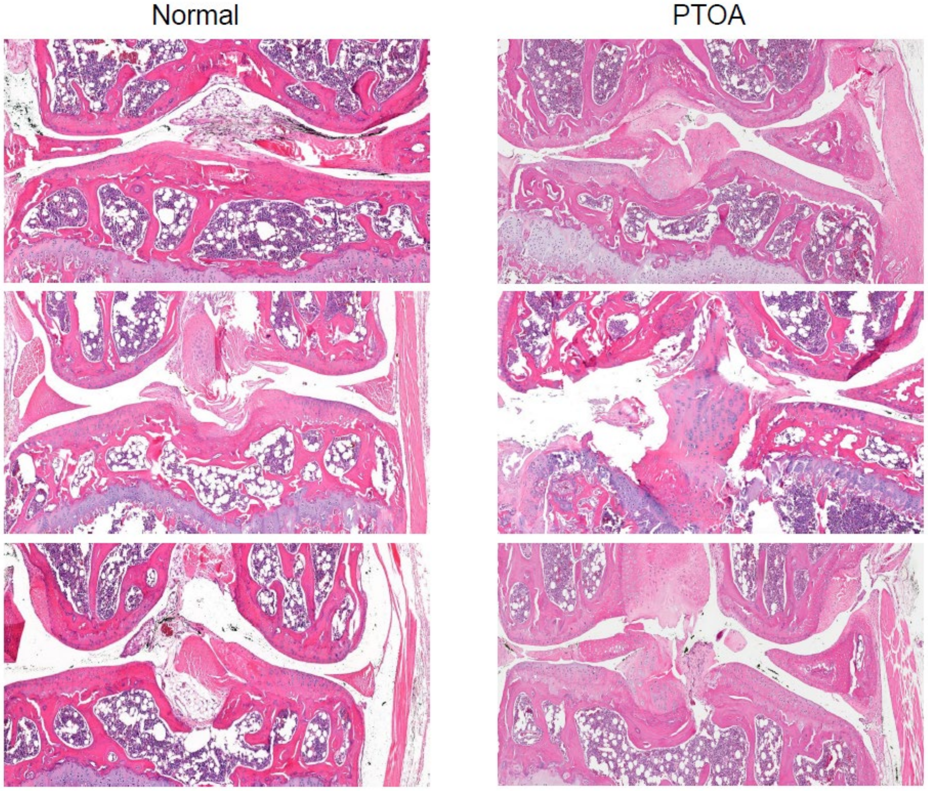

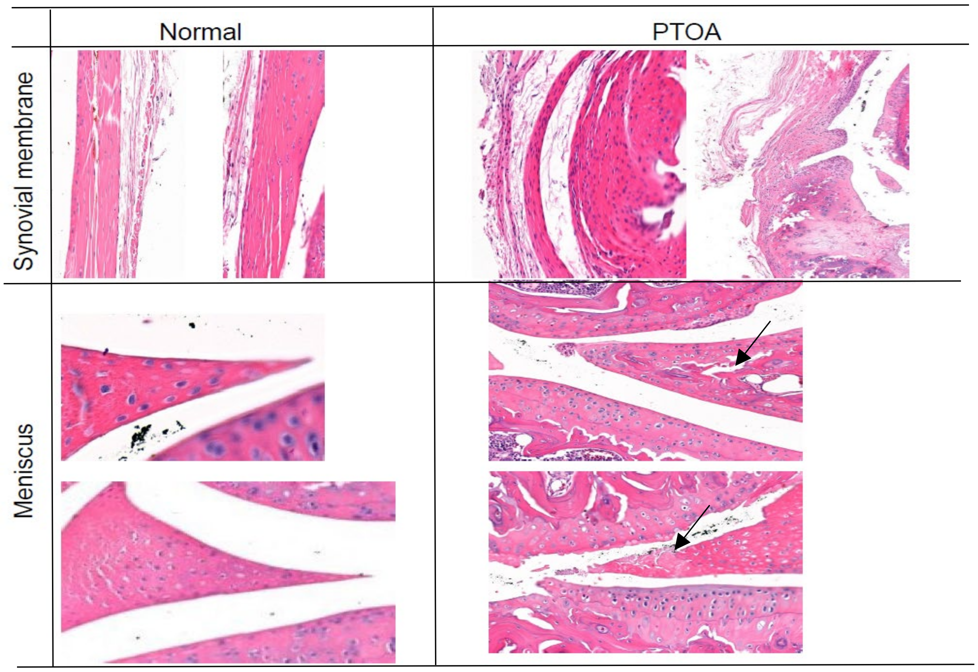

Blinded scoring result

CATEGORY		POINTS	Normal Slide1 MT Rvw1
**Hypocellularity**			
	Normal <5%	0	0
	Small decrease in chondrocytes <25%	1	
	Large decrease in chondrocytes 25-75%	2	
	No cells >75%	3	
**Safranin-O staining**			
	Normal	0	0
	Slightly reduced	1	
	Markedly reduced	2	
	No metachromatin stain	3	
**Surface regularity**			
	Smooth (>3/4)	0	0
	Moderate (1/2 to 3/4)	1	
	Irregular (1/4 to 1/2)	2	
	Severely irregular (<1/4)	3	
**Structure**			
	Normal / Tide mark(intact)	0	0
	Deformation (irregular or unclear tide mark)	1	
	Fibrillation / surface loss (>1/4)	2	
	Fissures/ Tide mark(crossed the line)	3	
	Erosion / loss cartilage (>1/2)	4	
	Complete disorganization	5	
**Sub Total**		**14**	0
**Total**		56	

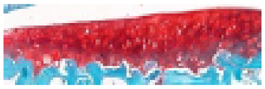			Normal Slide1 MF	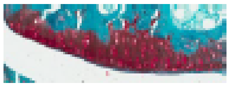			Normal Slide1 LT	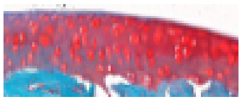	
Rvw2	Rvw3	Avg	Rvw1	Rvw2	Rvw3	Avg	Rvw1	Rvw2	Rvw3
0	0		0	0	0		0	0	0
0	0		0		0		0	0	0
				1					
0	0		0	0	0		0	1	0
0	0		0	0	1		0	0	0
0	0	0	0	1	1	0.666667	0	1	0
	stdv	0			stdv	0.57735			stdv

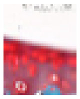

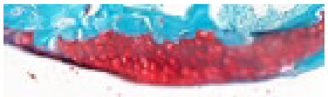

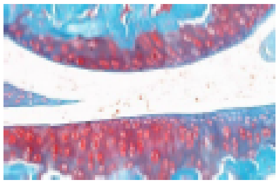

	Normal Slide1 LF				Trauma Slide1 MT				Trauma Slide1 MF
Avg	Rvw1	Rvw2	Rvw3	Avg	Rvw1	Rvw2	Rvw3	Avg	Rvw1
	0	0	0		0	0	1		0
	0	1	0		1	1	1		1
	0	0	0		0	0	0		0
	0	0	1		0	0	1		0
0.333333	0	1	1	0.666667	1	1	3	1.666667	1
0.57735			stdv	0.57735			stdv	1.154701	
			Avg Total	1.666667					

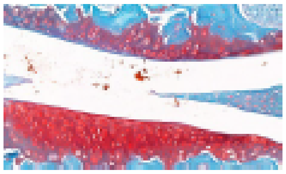

			Trauma Slide1				Trauma Slide1		
			LT				LF		
Rvw2	Rvw3	Avg	Rvw1	Rvw2	Rvw3	Avg	Rvw1	Rvw2	Rvw3
0	0		0	0	1		0	0	0
1	1		1	0	0		1	1	2
0	0		0	0	0		0	0	0
0	1		0	0	0		0	0	1
1	2	1.333333	1	0	1	0.666667	1	1	3
	stdv	0.57735			stdv	0.57735			stdv
									**Avg Total**

	PTOA<br>Slide1	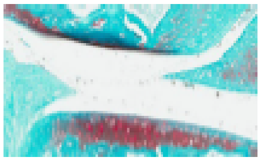			PTOA<br>Slide1				PTOA<br>Slide1
	MT				MF				LT
Avg	Rvw1	Rvw2	Rvw3	Avg	Rvw1	Rvw2	Rvw3	Avg	Rvw1
	0								
		1	1						1
					2	2			
							3		
	1	1							1
			2		2	2	2		
	0	0	0		0	0	0		
									1
					2	2			
							3		
		0							
	1		1						
									3
					4	4			
							5		
1.666667	2	2	4	2.666667	10	10	13	11	6
1.154701			stdv	1.154701			stdv	1.732051	
5.333333									

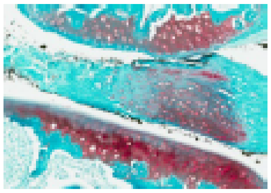

PTOA
Slide1

Normal
Slide2

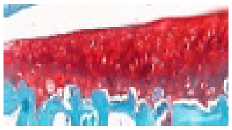

			LF				MT		
Rvw2	Rvw3	Avg	Rvw1	Rvw2	Rvw3	Avg	Rvw1	Rvw2	Rvw3
							0	0	0
1			1	1					
	2				2				
	1						0	0	0
2			2	2	2				
0	0						0	0	0
				2					
			3		3				
	0						0	0	0
1									
				2	2				
			3						
4	3	4.333333	9	7	9	8.333333	0	0	0
	stdv	1.527525			stdv	1.154701			stdv
					**Avg Total**	26.33333			

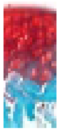	**Normal Slide2**	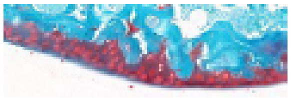			**Normal Slide2**	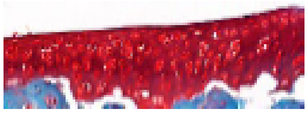			**Normal Slide2**
	MF				LT				LF
Avg	Rvw1	Rvw2	Rvw3	Avg	Rvw1	Rvw2	Rvw3	Avg	Rvw1
	0	0	0		0	0	0		0
	1	0	0		0	0	0		0
	0	0	0		0	0	0		0
	0	0	0		0	0	0		0
0	1	0	0	0.333333	0	0	0	0	0
0			stdv	0.57735			stdv	0	

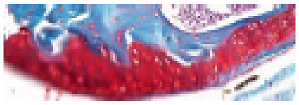

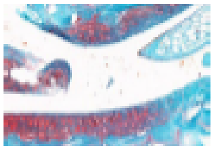

			Trauma Slide2				Trauma Slide2		
			MT				MF		
Rvw2	Rvw3	Avg	Rvw1	Rvw2	Rvw3	Avg	Rvw1	Rvw2	Rvw3
0	0		0		0				
				1			1	1	1
0	1		0				1	1	
				1	1				2
0	0		0	0	0				
							1	1	1
0	0		0	0	0				
							1		1
								2	
0	1	0.333333	0	2	1	1	4	5	5
	stdv	0.57735			stdv	1			stdv
	**Avg Total**	0.666667							

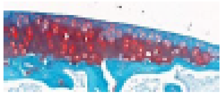

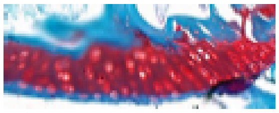

	**Trauma**				**Trauma**				**PTOA**
	**Slide2**				**Slide2**				**Slide2**
	LT				LF				MT
Avg	Rvw1	Rvw2	Rvw3	Avg	Rvw1	Rvw2	Rvw3	Avg	Rvw1
	0								
		1	1		1	1	1		1
									0
					1	1	1		
	1								
		2	2						
	0	0	0						0
						1			
					2		2		
		0	0						0
	1				1	1	1		
4.666667	2	3	3	2.666667	5	4	5	4.666667	1
0.57735			stdv	0.57735			stdv	0.57735	
							Avg Total	13	

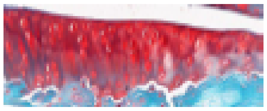

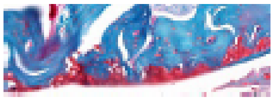

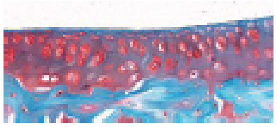

			PTOA Slide2				PTOA Slide2		
			MF				LT		
Rvw2	Rvw3	Avg	Rvw1	Rvw2	Rvw3	Avg	Rvw1	Rvw2	Rvw3
	0								
1							1	1	1
			2		2				
				3					
0									
	1						1	1	
			2	2	2				2
0	0						0	0	0
			2	2					
					3				
0							0		
	1							1	1
			4	4	4				
1	2	1.333333	10	11	11	10.66667	2	3	4
	stdv	0.57735			stdv	0.57735			stdv

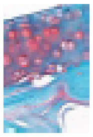

PTOA
Slide2

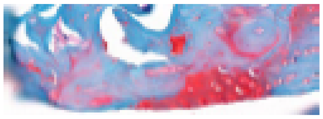

Normal
Slide3

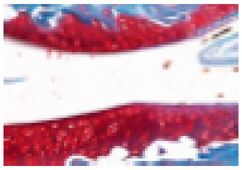

	LF					MT			
Avg	Rvw1	Rvw2	Rvw3	Avg		Rvw1	Rvw2	Rvw3	Avg
						0	0	0	
	2	2							
			3						
						0	0	0	
	2	2	2						
						0	0	0	
		1							
	2		2						
						0			
							1	1	
	4	4							
			5						
3	10	9	12	10.33333		0	1	1	0.666667
1			stdv	1.527525				stdv	0.57735
			**Avg Total**	25.33333					

**Normal Slide3**				**Normal Slide3**	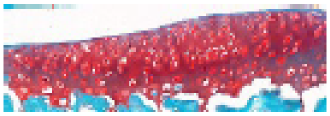			**Normal Slide3**	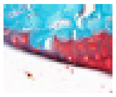
MF				LT				LF	
Rvw1	Rvw2	Rvw3	Avg	Rvw1	Rvw2	Rvw3	Avg	Rvw1	Rvw2
0	0	0		0	0	0		0	1
0	0	1		0	0	0		0	0
0	0	0		0	0	0		0	0
0	1	1		0	0	0		0	1
0	1	2	1	0	0	0	0	0	2
		stdv	1			stdv	0		

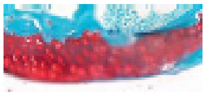			Trauma Slide3	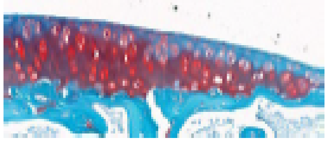			Trauma Slide3	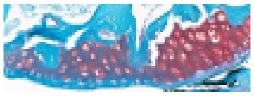	
			MT				MF		
Rvw3	Avg		Rvw1	Rvw2	Rvw3	Avg	Rvw1	Rvw2	Rvw3
1			0	1	1		1	1	2
1			1	1	2		2	2	2
0			0	0	0		2	1	2
1			1	1	1		3	2	3
3	1.666667		2	3	4	3	8	6	9
stdv	1.527525				stdv	1			stdv
Avg Total	3.333333								

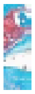

	Trauma Slide3 LT	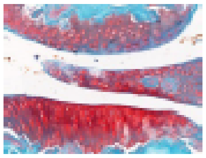			Trauma Slide3 LF					PTOA Slide3 MT 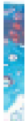
Avg	Rvw1	Rvw2	Rvw3	Avg	Rvw1	Rvw2	Rvw3	Avg		Rvw1
	0	1	0		0	1	0			1
	0	0	0		1	1	1			1
	0	0	0		0	0	0			0
	0	1	0		1	1	1			0
7.666667	0	2	0	0.666667	2	3	2	2.333333		2
1.527525			stdv	1.154701			stdv	0.57735		
							Avg Total	13.66667		

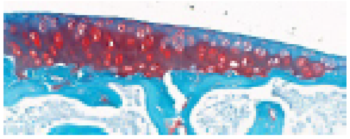

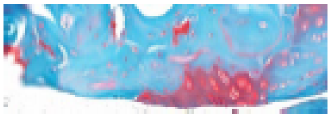

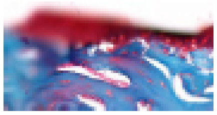

			**PTOA**				**PTOA**		
			**Slide3**				**Slide3**		
			MF				LT		
Rvw2	Rvw3	Avg	Rvw1	Rvw2	Rvw3	Avg	Rvw1	Rvw2	Rvw3
1	1								
			2				2	2	2
				3	3				
1									
	2		2	2			2	2	2
					3				
0	0								
			2						
				3	3		3	3	3
0									
	1								
			4	4	4		4		4
								5	
2	4	2.666667	10	12	13	11.66667	11	12	11
	stdv	1.154701			stdv	1.527525			stdv

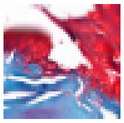

PTOA
Slide3

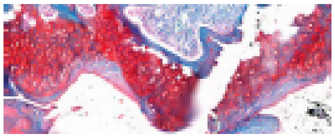

LF

Avg	Rvw1	Rvw2	Rvw3	Avg
	2	2	2	
	1			
		2	2	
	2		2	
		3		
	4			
		5	5	
11.33333	9	12	11	10.66667
0.57735			stdv	1.527525
			**Avg Total**	36.33333

	slide1	slide2	slide3	Avg	stdv	p value<br>t N v Trau	t N v PTOA	t Trau v PTOA
Normal	1.666667	0.666667	3.333333	1.888889	1.347151	0.039357	0.005045	0.014102
Trauma	5.333333	13	13.66667	10.66667	4.630815			
PTOA	26.33333	25.33333	36.33333	29.33333	6.082763			
